# Removal of One-Half of the Lower Maxilla for Secondary Cancerous Manifestation

**Published:** 1852-10

**Authors:** 


### ARTICLE XIX.

### *Removal of one-half of the Lower Maxilla for Secondary Cancerous Manifestation.*

The removal of either the upper or lower maxilla has now become an established operation in surgery, many successful cases have been recorded, and the next generation of surgeons will doubtless feel emboldened, and perform the operation with great confidence. It should, however, not be concealed that cases occur which do not terminate successfully, and that the greatest caution will always be necessary in the selection of the patients which are most fit for operation—with whom, namely, a renewed development of the disease is not to be feared, and where the subject possesses sufficient stamina to resist the effects of a procedure in which chloroform can only partially be taken advantage of.

The patient upon whom Mr. Hancock operated, a few days ago, in Charing Cross Hospital, was suffering from the recurrence of cancerous disease just below and upon the lower jaw, on the left side, in which locality a fungous growth, about three inches in diameter, circular, depressed in the centre, and malignant-looking, had presented itself. The man is forty-three years of age, following the occupation of cabman, and it appears that, about a year and a half ago, he received a blow on the lower lip, from a horse's bit, the sharp end of which pierced the soft parts, and forced a portion of the lip into the mouth, which portion, according to his statement, he bit off. After some time, a chasm was left in the lip, which latter, after proper treatment, resumed very nearly its healthy condition. Two months afterwards, the part ulcerated upon a bilious attack, and has continued to do so up to the present time. The patient further states, that upon his first going out after his bilious attack, he felt a "kernel," as he calls it, in the neck, immediately under the lower jaw, on the left side. This gradually enlarged for the space of four months, in spite of various modes of treatment, and about five weeks before admission, it began to ulcerate. There is severe lancinating pain in the tumor, which has finally assumed a fungous, cancerous aspect, and is discharging sanious matter. The patient's habits have been temperate, and he was never effected with the venereal disease.

It was considered that the bone was implicated, and that the only chance of doing the man any permanent good was to remove a portion of the lower jaw. Chloroform was administered, and the first incision ran from over the malar bone to the commissure, and the second from the same point round and below the tumor, to about an inch and a half beyond the symphysis. As the tumor implicated a large portion of the skin, much of the latter was necessarily sacrificed; the flaps were dissected upwards and downwards, the vessels tied, and the bone divided, first to the right of the symphysis, and secondly, close to the coronoid process, both with saw and forceps. The bone being removed, it was found that the submaxillary gland was involved in the disease, as well as a portion of the surrounding muscular tissue. The soft parts were now rapidly excised, and as they were composed of all of the muscles which connect the tongue with the jaw, the chasm became very deep, and the tongue lost most of its support on the left side. This organ was carefully held out with a thread passed through its root, by which precaution asphyxia by the falling of the tongue upon the epiglottis was prevented. The effects of chloroform were partially kept up all this while, and the numerous vessels quickly tied, but the loss of blood was unavoidably great. The gap left on the side of the face could evidently be closed up only by transplantation of soft parts, but this was postponed to a further operation.

The examination of the pathological specimen proved that the tumor was firmly attached to the bone, but that it had as yet implicated the periosteum only. We shall give an idea of the extent of jaw removed, by stating that the gums contained ten teeth.—*London Lancet.*

